# Seismic collapse risk of RC-timber hybrid building with different energy dissipation connections considering NBCC 2020 hazard

**DOI:** 10.1186/s43065-022-00061-6

**Published:** 2022-11-06

**Authors:** Ikenna Odikamnoro, Prakash S. Badal, Henry Burton, Solomon Tesfamariam

**Affiliations:** 1 School of Engineering, University of British Columbia, Kelowna, V1V 1V7 BC Canada; 2 Department of Civil and Environmental Engineering, University of California, Los Angeles, CA 90095 USA

**Keywords:** Hybrid system, Seismic hazard update, Cross-laminated timber wall, Energy dissipation connections, National Building Code of Canada (NBCC)

## Abstract

The 2020 National Building Code of Canada (NBCC) seismic hazard model (SHM) marks a comprehensive update over its predecessor (NBCC 2015). For different regions in Canada, this will have an impact on the design of new buildings and performance assessment of existing ones. In the present study, a recently developed hybrid building system with reinforced concrete (RC) moment-resisting frames and cross-laminated timber (CLT) infills is assessed for its seismic performance against the latest SHM. The six-story RC-CLT hybrid system, designed using the direct displacement-based method, is located in Vancouver, Canada. Along with very high seismicity, southwestern British Columbia is characterized by complex seismotectonics, consisting of subduction, shallow crustal, and in-slab faulting mechanisms. A hazard-consistent set of 40 ground motion pairs is selected from the PEER and KiK-net databases, and used to estimate the building’s seismic performance. The effects of using steel slit dampers (associated with large hysteresis loops) and flag-shaped energy dissipators (associated with the recentering capability) are investigated. The results indicate that the hybrid system has good seismic performance with a probability of collapse of 2–3% at the 2475-year return period shaking intensity. The hybrid building with steel slit dampers exhibits a collapse margin ratio of 2.8, which increases to 3.5–3.6 when flag-shaped dissipators are used. The flag-shaped dissipators are found to significantly reduce the residual drift of the hybrid building. Additionally, the seismic performance of the hybrid building equipped with flag-shaped dissipators is found to improve marginally when the recentering ratio is increased.

## Introduction

Buildings have become a key point of focus in establishing sustainable development goals meant to address the growing challenge of climate change [1]. With rapid population growth in urban areas, non-traditional buildings such as those with hybrid structural systems are central to the development of sustainable infrastructure. Over the past couple of decades, concrete and steel have become the materials of choice for the construction of civil infrastructure. With a robust design methodology, traditional concrete and steel structures have dominated the construction market. However, with the focus shifting to environmentally friendly and lightweight structures, timber is fast becoming a viable construction material [2]. One of the benefits of timber construction in seismic zones is its light weight, which attracts smaller inertial forces when compared to concrete or steel structures with the same size and complexity. Aside from being used as a stand-alone material for building structural systems as permitted by the various design codes, several hybrid-structural systems using timber have been investigated, e.g. [3–9].

Prescriptive design standards, based on a force-based methodology, utilize reduction factors to account for inherent non-linearity in the structural systems [10]. Several researchers have worked to develop the reduction factors for different hybrid-structural systems [11, 12]. However, existing design codes do not provide the reduction factors for emerging hybrid-structural systems, consequently, force-based design cannot readily be implemented. In an attempt to provide a more robust, accurate, and reliable design approach, the performance-based design methodology was developed [13–15]. One variation of that methodology is direct displacement-based design (DDBD) [16]. Starting with the design of simple concrete multi-span bridges and building frames [17], the DDBD has been extensively applied to traditional and novel systems. Examples include structures with passive energy dissipating devices [18], steel-timber hybrid buildings [6], frame-wall structures [19, 20], frame-wall structures with dissipators [21], structures with seismic isolation systems [22], steel and RC moment-resisting frames [23–25], traditional light-frame timber structures [26], structures with flexible bases [27], tall hybrid timber buildings [26], self-centering buckling-restrained braced frame structures [25, 28], base-isolated building structures [29], dual systems [30], and structures with visco-elastic dampers [31]. Recent developments have paved the way for high-rise building design using the DDBD by addressing issues of the displacement profile [32], higher modes, and P-Delta effects [24, 25].

An integral part of the DDBD method is estimating the equivalent viscous damping (EVD) for different structural systems [33, 34]. Over the years, different researchers have sought to establish expressions for determining the EVD of different structural systems, e.g., Table 1. This was generally achieved by using results from a physical experiment to calibrate a numerical model, and then EVD-ductility relationships are developed for a large number of numerical models using an area-based approach. The ductility, $\mu$, is defined as the ratio of the maximum displacement to the yield displacement. In recent years, EVD expressions for hybrid buildings using timber and different dissipation mechanisms have been developed [6, 35–40]. These studies will facilitate the design and widespread acceptance of hybrid timber buildings. The present study adopts an RC-timber hybrid building designed using the DDBD approach [8]. A maximum story drift ratio ($IDR_{max}$) of 2.5% that corresponds to the collapse prevention limit state is targeted. The EVD for the RC-timber hybrid system was developed by Nielsen and Imbeault [41]. The hybrid building system utilizes the high strength and stiffness characteristics of the CLT to reduce the induced displacement of the structure and the energy dissipating characteristics of the hysteretic connectors [8, 42].

**Table 1:** Equivalent viscous damping $(\xi _{eq})$ (%) and ductility $(\mu )$ relationships for different structural systems

Description	EVD Expression $(\xi _{eq})$	Parameter definitions
Precast concrete walls and frames [43]	$5+\frac{25}{\pi }\left( 1-\frac{1}{\sqrt{\mu }}\right)$	
RC frame [44]	$\max (4.7, -0.4\mu ^2 + 7.1\mu - 2)$	
RC frame [34]	$\xi _0+\frac{8}{\pi }\left( 5-\frac{6}{\mu ^{1.8}}+0.2\mu ^{1.8}\right)$	$\xi _0$: Initial elastic damping
BRB-RC frame [45]	$5+\left( \frac{150(\mu -1)(1-0.14)}{(\mu -0.14\mu +0.14\mu ^2)\pi }\right)$	
Steel-braced RC frame [34]	$\xi _0+\frac{70}{\pi }\left( 1-\frac{43}{\mu ^4}-\frac{4.7}{10^5}\mu ^4\right) ; \mu \ge 3$	$\xi _0$: Initial elastic damping
Infilled-RC frame [46]	$\xi _0+80.4\left( \frac{\mu +0.83}{\mu \pi }\right)$; $\xi _0+80.4\left( \frac{\mu +0.05}{\mu \pi }\right)$	$\xi _0$: Initial elastic damping
Steel members [47]	$\xi _0+a\left( 1- \frac{1}{\mu ^b}\right) T^c$	$\xi _0$: Initial elastic damping, *a*, *b*, and *c*: constants, *T*: Period
Hybrid-timber [40]	$5+\frac{62\times \lambda ^{0.4}}{\pi }\left( 1-\frac{1}{\sqrt{\mu }}\right)$	$\lambda =H/B$: slenderness ratio, *H*: height of frame, *B*: width of the frame
Steel frame with CLT infill [33]	$\xi _0 + C\left( \frac{\mu -1}{\mu \pi }\right)$	*C*: constant

While it is important that sustainable alternatives to traditional structural systems be developed, attention must be paid to their seismic collapse risk to ensure adequate life safety performance. As such, seismic collapse risk assessment of buildings has gained attention [48]. In the present study, the collapse capacity of the RC-CLT building reported in [8] is assessed using nonlinear time-history analysis. The effects of the energy dissipation devices have been studied by considering an alternative flag-shaped dissipator which has gained widespread interest in the last two decades [49–54]. The hybrid building reported in Tesfamariam et al. [8] was designed with the NBCC 2015 [55] seismic hazard. With the newly released updated NBCC 2020 [56] hazard model, it is postulated that substantial changes in the seismic hazard will have an impact on the design of new buildings and assessment of existing ones, especially in regions with very high seismicity, such as southwestern British Columbia. The seismic hazard of the selected Vancouver, British Columbia site, is affected by the interaction of three tectonic regimes, viz., Subduction, Shallow Crustal, and In-slab. A hazard-consistent 40-paired ground motion records, representing the complex seismicity of Vancouver, is selected for nonlinear response history analysis of the hybrid building system.

The present study has three primary contributions—(i) a rigorous ground motion record selection consistent with the complex seismicity of Southwestern British Columbia is performed. The record selection follows the latest seismic hazard model per NBCC 2020 [56]. (ii) The seismic collapse assessment of an innovative RC-CLT building system designed using DDBD is assessed, and (iii) the effects of alternative energy dissipators on collapse capacity and residual deformation are investigated.

## Design details of the RC-CLT hybrid building with steel slit dampers

A regular 6-story, 3-bay RC moment resisting frame with CLT infill designed by Tesfamariam et al. [8] has been considered for the seismic performance assessment. The building is 24 m $\times$ 42 m in plan with the longer direction having 7 bays each with a length of 6 m. Figure 2 shows the elevation along the shorter and more critical direction. The CLT infill is a three-ply panel that is 99 mm thick, comprised of three 33 mm thick laminae that are fused together. The CLT infill was designed with some clearance to the RC frame to avoid shear (brittle) failure in the RC columns due to their interaction [57]. Slit dampers [42] at the top of the CLT infill were provided as an additional energy dissipation mechanism. The steel slit dampers are associated with large hysteretic loops leading to high energy-dissipation capacity. Given the novel structural system, the RC-CLT building was designed using the DDBD. The target was set to a maximum inter-story drift $IDR_{max}$ of 2.5% corresponding to 2% probability of exceedance in 50 years, for the NBCC 2015 hazard level [55].

**Fig. 1:**
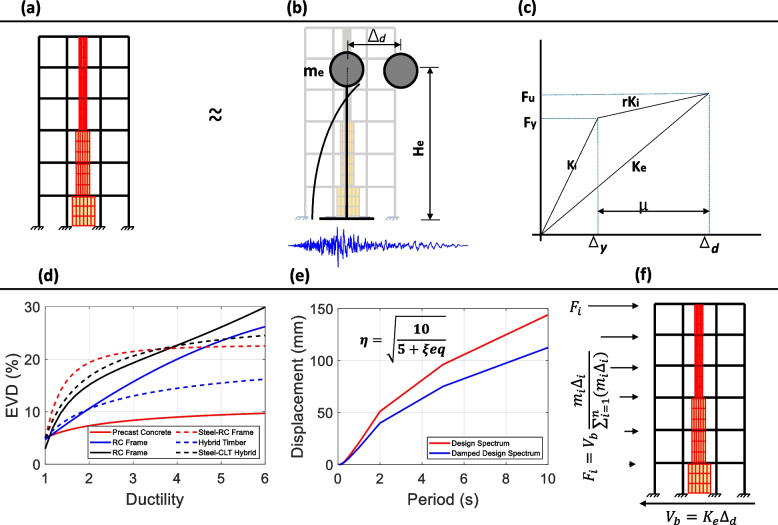
DDBD procedure: **a** Structure description **b** Equivalent SDOF system and its displaced shape **c** Bi-linear force-displacement relationship of the SDOF system **d** EVD-$\mu$ relationship for the different structural types **e** Displacement response spectra **f** Distribution of the seismic design load along the building height

Figure 1 summarizes the DDBD procedure as follows:

1. Develop the design displacement profile.
2. Determine the characteristics of the equivalent single degree of freedom system.
3. Determine the design ductility.
4. Estimate the Equivalent Viscous Damping
5. Determine the effective period, stiffness and base shear.
6. Perform structural analysis and member design.
7. Calculate the stiffness of each story.
8. Calculate the equivalent stiffness of the CLT and dampers.
9. Calculate the number of dampers required for each story.
10. Design and validate the structure.

**Fig. 2:**
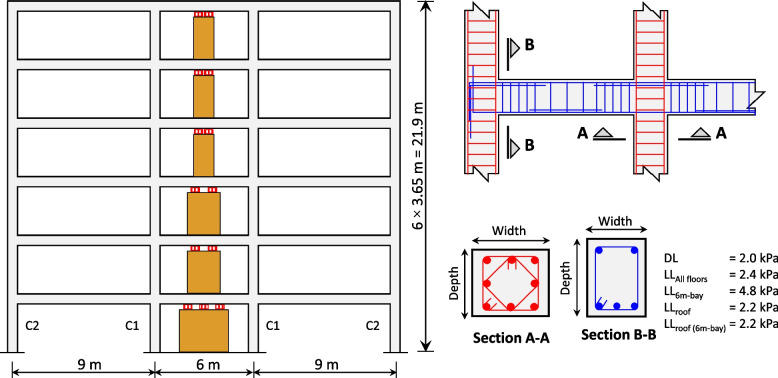
Details of the six-story RC-CLT hybrid building [8]

A more detailed DDBD procedure for the RC-timber hybrid building is described in Tesfamariam et al. [8]. The elevation of the hybrid building is shown in Fig. 2. It is noted that the length of the CLT infill and the number of slit dissipators are optimized during the design. A summary of the results from the design is given in Table 2. All beams are 450 mm $\times$ 350 mm in size. The design is governed by the connection with the slit damper and gravity loads on each floor.

**Table 2:** Design summary for 6-story RC-CLT hybrid building

Story	Column size (mm^2^)	$\rho _{b,top}$	$\rho _{b,bot}$	$\rho _{C1}$	$\rho _{C2}$	No. of dampers	$L_{CLT}$ (m)
6	300$\times$300	2-20M	2-20M	4-25M	4-20M	2	1
5	300$\times$300	2-20M	2-20M	4-25M	4-20M	2	1
4	300$\times$300	2-20M	2-20M	6-25M	6-20M	2	1
3	350$\times$350	2-20M	3-20M	6-25M	6-20M	2	2
2	350$\times$350	2-20M	3-20M	8-25M	6-25M	2	2
1	350$\times$350	2-20M	3-20M	8-25M	6-25M	3	3

$\rho _{b,top}, \rho _{b, bot}$: longitudinal reinforcement at top and bottom of beams; $\rho _{C1}, \rho _{C2}$: longitudinal reinforcement in column C1 and C2, respectively; $L_{CLT}$: length of CLT infill wall

## Nonlinear structural modeling of the RC-CLT hybrid building

**Fig. 3:**
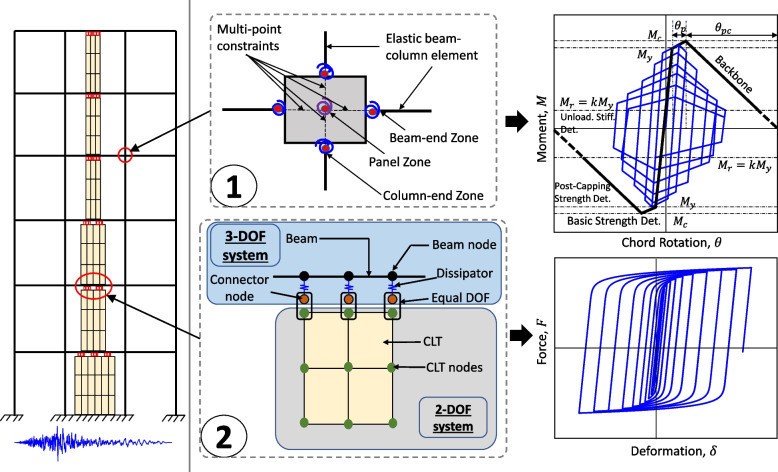
Schematic sub-assembly of the nonlinear structural model for RC-CLT hybrid building system

A two-dimensional nonlinear model was considered to be appropriate due to the regularity of the hybrid building. Figure 3 provides a schematic representation of the key elements of the analytical model. A concentrated plasticity model is used to capture the non-linearity in the RC frame. Marker 1 shows the modeling details of the RC frame whereas Marker 2 describes the details of the RC-CLT dissipator connections. Different structural components are provided in the following subsections. The P-Delta effects are captured using a leaning column [58]. 5% Rayleigh damping has been considered in the first and the third fundamental mode for all linear elastic elements. Damping in the nonlinear elements has not been modeled as recommended in the literature [59–61]. The eigenvalue analysis of the frame resulted in first, second, and third mode periods of 1.17, 0.40, and 0.25 seconds, respectively.

### CLT modeling

The three-ply 99 mm thick CLT infill panel is modeled using quad elements with a bilinear isoparametric finite element formulation. The CLT infill is formulated assuming a plane stress condition due to the minimal out-of-the-plane stresses. Because of the connection detail and design of the hybrid building, the assigned nDMaterial for the CLT elements are elastic with a modulus *E*, of 9.5 kN/mm^2^, and a Poisson’s ratio of 0.16. The CLT and RC frame are connected using steel-slit dampers. The stiffness of the CLT, $K_{CLT}$, is approximated as follows [62]:

1 $$\begin{aligned} K_{CLT} = \frac{1}{\frac{2h^3}{3EAL}+\frac{h}{1000G}+\frac{hd}{Lf}}, \end{aligned}$$

where *h*, *E*, *A*, *L*, *G*, *d*, and *f* represent the CLT height, elastic modulus, cross-sectional area, wall length, shear modulus, thickness, and bearing capacity factor per CSA O86 [63], respectively.

### RC frame modeling

The modeling details of a typical RC beam-column joint are shown with Marker 1 in Fig. 3. The RC beam-column joints are designed and detailed to conform with the highest ductility class specified in the Canadian standard [55]. Thus, flexural yielding is expected to precede shear failure. The RC members are modeled using the hysteretic rules defined by the Ibarra-Medina-Krawinkler (IMK) model [64], which captures strength and stiffness degradation. The key points on the backbone curve and cyclic deterioration constants are based on the semi-empirical expressions developed in the literature [65, 66]. The multi-point constraints for the RC joints are modeled using the joint2D element that incorporates a diagonal compression strut mechanism [67] and a shear panel is used for the joint deformation.

### RC-CLT-Damper modeling

The modeling details of the RC-CLT joint including the slit dampers on top of the CLT infill are shown with Marker 2 in Fig. 3. The beam-column elements have 3 degrees of freedom ($u_x$, $u_y$, $r_z$) and the quad element has two ($u_x$, $u_y$). The two elements with different numbers of degrees of freedom are linked using *connector nodes* which are constrained using the equalDOF command. A twoNodeLink element is used between the connector and RC beam nodes. The bottom of the CLT infill is anchored to the RC beam by constraining the quad elements at the base of each floor. Due to the dominance of flexural behavior ($r_z$), the chord rotation is used as the deformation measure for the RC members, whereas the displacement is used as the deformation measure for the energy dissipators due to their point action along the translational direction ($u_x$).

**Fig. 4:**
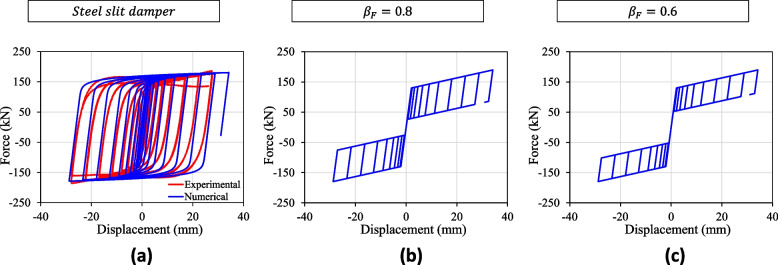
Cyclic responses of the **a** steel slit damper and **b**,**c** flag-shaped dampers with **b** $\beta _F = 0.8$ and **c** $\beta _F = 0.6$ obtained using OpenSees. For steel slit dampers, the comparison of experimental hysteretic response is also presented

Figure 4a shows an example calibration of the hysteretic response of the slit damper using OpenSees [68], which is modeled using the RambergOsgoodSteel uniaxial material. The experimental data are taken from Lee et al. [42]. The calibrated parameters for the RambergOsgoodSteel material are: the yield strength $F_y= 140$ kN, initial elastic modulus $E_0 = 70$ kN/mm, yield offset $a = 0.002$, and the parameter to control the transition from elastic to plastic response $n= 13$. Similarly, Fig. 4b–c show the numerical response of the flag-shaped dampers. Two recentering ratio values, $\beta _F = 0.8$ and $\beta _F = 0.6$, have been considered. These values are based on relevant experimental studies [39]. The flag-shaped dampers are modeled using the SelfCentering uniaxial material in OpenSees. In the absence of experimental results for the flag-shaped dampers of equivalent strength, their key parameters are selected such that the peak strength and deformation capacity closely resemble that of the steel slit dampers. Nonlinear static analyses of these systems are used to confirm this design choice. The adopted parameters for flag-shaped dissipators are: the forward activation strength $F_{a} = 130$ kN, initial stiffness $k_1 = 61.9$ kN/mm (corresponding to 2.1 mm of deformation at activation strength), and post-activation stiffness $k_2 = 0.03 k_1$.

## Seismic hazard and GM selection per NBCC 2020

### Seismic hazard

**Fig. 5:**
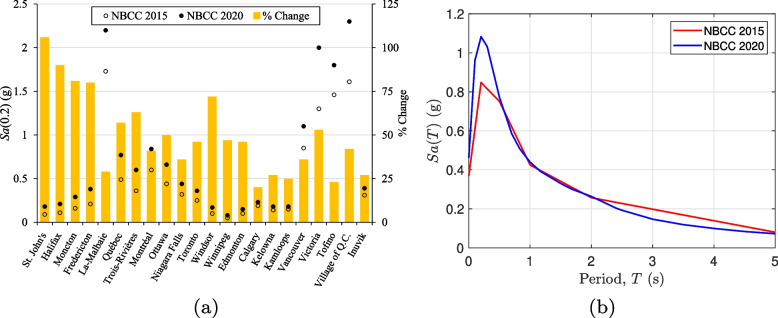
**a** Change in seismic hazard from NBCC 2015 to NBCC 2020 for selected locations in Canada, from east to west. Intensity measure- *Sa*(0.2) ; Return Period- 2475 year; Site Class- C ($V_{s30} = 450$ m/s). **b** Response spectrum for Vancouver based on the NBCC 2015 and NBCC 2020 seismic hazard models

**Fig. 6:**
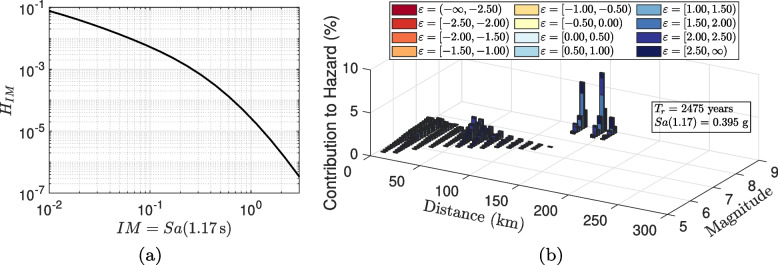
PSHA results based on the NBCC 2020 seismic hazard model. **a** Seismic hazard curve and **b** deaggregation based on $Sa(1.17\ \text {s})$ for the Vancouver site

The 6th generation seismic hazard model (SHM6) described in the NBCC 2020 [56] represents a comprehensive change over the NBCC 2015 [55]. Figure 5a shows the change in short period seismic hazard for several locations in Canada, from East to West. Such an increment of 25–50% is expected to impact the design and performance assessment of Canadian buildings, especially in regions with very high seismicity such as southwestern British Columbia. Figure 5b shows the response spectrum for Vancouver corresponding to the 2% in 50 year return period. However, it is noted that for the period of interest in the present study (first natural period of the CLT-RC hybrid building, $T_1 = 1.17 \text{ s}$), the two hazard models incidentally have approximately the same spectral acceleration value. Figure 6a shows the hazard hazard curve for the Vancouver site ($49^\circ 15' 00''$ N, $123^\circ 7' 12''$ W) with an average shear wave velocity to 30 m depth, $V_{s30} =$ 450 m/s. The hazard curve is shown for the intensity measure, $Sa(1.17 \text { s})$ i.e., the spectral acceleration corresponding to the first mode. The deaggregation results for $Sa(1.17 \text { s})$ corresponding to a 2475 year return period is shown in Fig. 6b. Three contributing tectonic regimes are considered for southwestern British Columbia [69, 70]. For the present hazard, their contributions are 41% for Active Shallow Crust, 32% for Subduction Interface, and 27% for In-Slab (source id- IntraSlab55).

### Hazard-consistent ground motion selection

The ground motion selection is conducted to represent the complex tectonic feature of southwestern British Columbia. A series of site- and structure-specific conditional spectra (CS) is considered. Specific to a tectonic regime, the CS includes the mean (known as, conditional mean spectrum, CMS) and covariance between different spectral ordinates [71]. The CMS accounts for the conditionality of spectral ordinates by defining the parameter $\varepsilon$, as follows:

2 $$\begin{aligned} \varepsilon (T^*) = \frac{\ln S{a}(T^*) - \mu _{\ln Sa(T^*)}}{\sigma _{\ln Sa(T^*)}}, \end{aligned}$$

where $S{a}(\cdot )$ is the spectral acceleration; $T^*$ is the conditioning period; and $\mu _{\ln Sa(\cdot )}$ and $\sigma _{\ln Sa(\cdot )}$ are obtained from a suitable ground motion model for deaggaregted magnitude-distance $(\overline{M}, \overline{R})$. Further, the CMS is expressed as:

3 $$\begin{aligned} \ln [Sa(T)|Sa(T^*)] = \mu _{\ln Sa(T)} + \rho _{\varepsilon (T),\varepsilon (T^*)} \sigma _{\ln Sa(T)} \varepsilon (T^*), \end{aligned}$$

where $\rho _{\varepsilon (T),\varepsilon (T^*)}$ is the correlation coefficient between *Sa*(*T*) and $Sa(T^*)$.

Table 3 shows the modal earthquake tuples $(\overline{M}, \overline{R})$ with the highest contribution for each tectonic regime using SHM6. The corresponding deaggregation was shown earlier in Fig. 6b. The table also lists the spectral shape at 1.17 s for each tectonic regime. The ground motion models for each tectonic regime is selected from the corresponding logic tree of the tectonic regime used during Probabilistic Seismic Hazard Analysis (PSHA).

**Table 3:** Spectral value and deaggregation tuples for contributing tectonic regime for Vancouver site and $Sa(1.17 \text {s})$ corresponding to a return period of 2475 years

Tectonic Regime	Contribution to Hazard	$\overline{M}$	$\overline{R}$ (km)	$\overline{\varepsilon }(1.17\ \text {s})$	Ground Motion Model
Active Shallow Crustal	41%	7.35	15	0.777	CB14 [72]
Subduction Interface	32%	8.85	145	1.797	BCH16 [73]
Intra-Slab	27%	7.15	55	1.047	AB03-Sub [74]

**Fig. 7:**
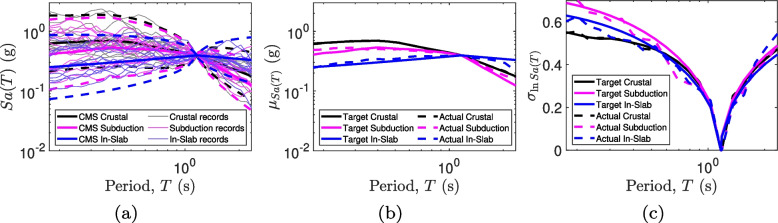
Scaled selected records targeted at the hazard and deaggregation corresponding to the Vancouver site. **a** Pseudo response spectra of individual records. Thick broken lines mark the 97.5%ile and 2.5%ile values, **b** target conditional mean for the three tectonic regimes and **c** target log-standard deviation for the conditional spectra. The conditioning period is 1.17 s

Figure 7 shows the results of the ground motion selection based on the target CS. The number of records from each tectonic regime is proportional to its contribution to the site hazard. Thus, there are 16 Shallow Crustal, 13 Subduction Interface, and 11 In-Slab records. The NGA West-2 database [75] has been used for crustal and in-slab records, whereas the KiK-net database [76] has been used for the subduction records. Site classification and flat-files for each database are available in the literature [77, 78]. The period range of interest is based on the NBCC guidelines [69, 79]. The upper bound of the period range, $T_{max}$ is defined as $\max (2T_1, 1.5\text { s})$, where $T_1$ is the period of vibration in the first mode. The lower bound of the period range, $T_{min}$ is defined as $\min (0.15T_1, T_{90\%})$, where $T_{90\%}$ is the lowest period of vibration that achieves a cumulative mass participation of 90%. For the RC-CLT building, the 0.25 second period corresponds to the third mode. Thus, the period range of interest is [0.18, 2.34]. A computationally-efficient algorithm is employed to select the ground motion suite [80]. The scaling factor is limited to 5 [69]. Further, a restriction of $R_{JB} > 20 \text { km}$ is imposed to exclude near-field records. A maximum magnitude difference of 1.5 from the modal magnitude is allowed. The $V_{s30}$ values are between 300 and 600 m/s. Figure 7b–c show the target and achieved mean and dispersion of the three conditional spectra. An excellent match reflects the versatility of the considered database and selection algorithm.

## Results

### Pushover analysis

**Fig. 8:**
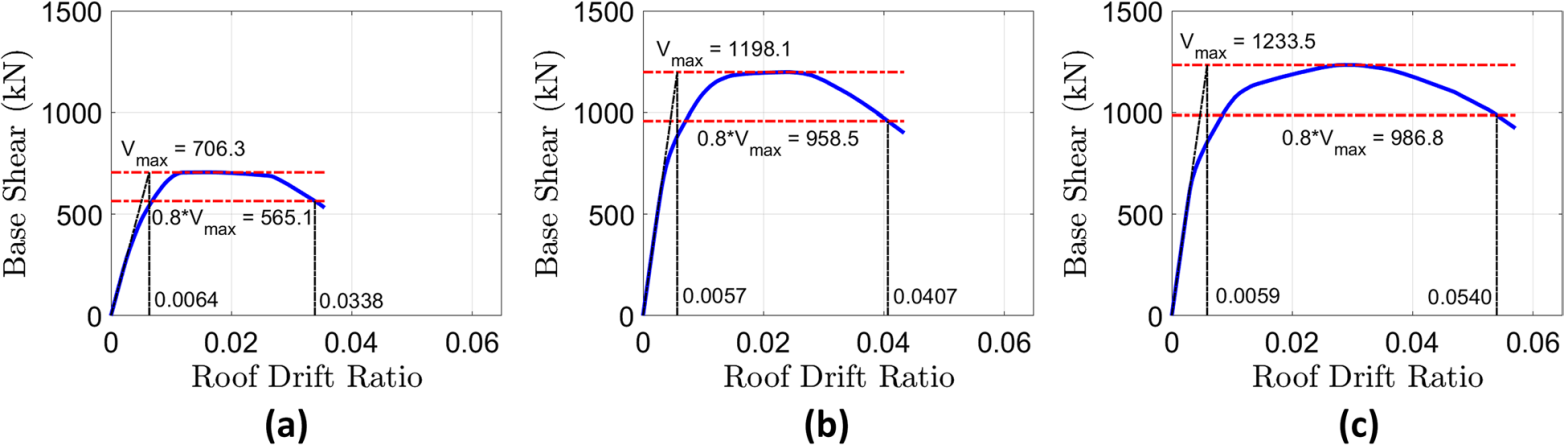
Pushover curves for the **a** 6-story bare RC frame, **b** RC-CLT hybrid building with steel slit dampers, and **c** RC-CLT hybrid building with flag-shaped dissipators

Figures 8b–c show the pushover curves for the RC-CLT hybrid frame with slit and flag-shaped dampers, respectively. The pushover curves for the latter with $\beta _F = 0.8$ and $\beta _F = 0.6$ are identical due to the common backbone curve of both flag-shaped dissipators. The effect of the recentering ratio is captured in the dynamic analysis, which is presented in the subsequent sections. The pushover curves of hybrid frames are compared with the bare RC frame (Fig. 8a) to observe the effects of the CLT infill and energy dissipators on the nonlinear static response. For both types of dissipators, a significant increase of $\approx~70\%$ is observed in the maximum base shear. While the yield displacements of the hybrid buildings are smaller than that for the bare frame, the maximum deformation capacity (corresponding to a base shear reduction to 80% of the maximum) increases. Thus, a combination of CLT infill with energy dissipators enhances the strength as well as the drift capacity of the building. The reduction in yield drift is attributed to the presence of the stiffer CLT infills, whereas the higher ultimate deformation is attributed to the high deformability of steel slit and flag-shaped dampers. The nonlinear static capacity of the hybrid frame with flag-shaped dissipators is similar to the frame with slit dampers. Since the design of frames is based on static analysis, this similarity confirms the equivalence of the design using both types of dissipators. The marginal difference in the inelastic capacity of the two dissipators is attributed to the discrete nature of the design process.

### Incremental dynamic analysis

**Fig. 9:**
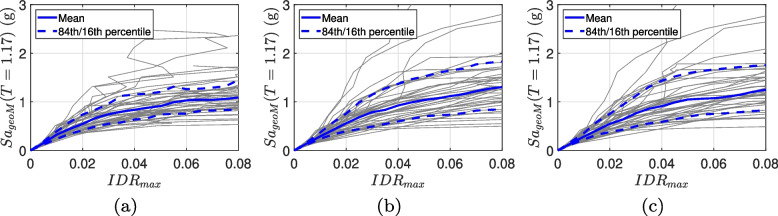
IDA curves for the 6-story RC-CLT hybrid building with **a** steel slit dampers, and with RC-CLT hybrid building with flag-shaped dissipators having **b** $\beta _F = 0.8$, and (c) $\beta _F = 0.6$ obtained using 40 pairs of NBCC 2020-compliant site-specific ground motion records. $IDR_{max}$ represents the maximum interstory drift ratio

The seismic performance of the RC-CLT hybrid system with different energy dissipators is assessed using incremental dynamic analysis (IDA) [81]. Under IDA, increasingly scaled ground motion records are applied to the building until its collapse. The statistical distribution of the intensity measure values for each ground motion record in the suite is used to derive the seismic fragility function. Due to the unique seismicity of Southwestern British Columbia, a rigorous ground motion selection method is adopted. As discussed earlier, a suite of 40 pairs of ground motion records is selected to represent the three tectonic regimes that affect Vancouver, British Columbia. Figure 9 shows the IDA curves for RC-CLT buildings with three design configurations of energy dissipators, viz., one slit damper and two flag-shaped dissipators with recentering ratio, $\beta _F = 0.8$ and $\beta _F = 0.6$. The figure also shows the 84th and 16th percentile IDA curves. The median collapse capacity of buildings with flag-shaped dissipators is observed to be the highest. This may be attributed to the recentering capacity of flag-shaped dissipators, which result in relatively smaller nonlinear deformations. It is noted that the scaling of the ground motion records for IDA maintains the same spectral shape at different intensities, which may not be a true representation of the hazard. Multiple stripe analysis targeting conditional spectra at different intensities can alleviate this issue [82, 83].

### Fragility assessment

**Fig. 10:**
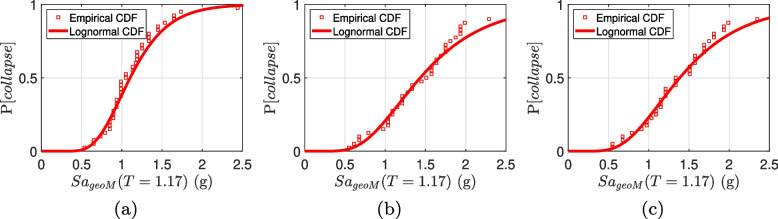
Collapse fragility curves for the 6-story RC-CLT hybrid building with **a** steel slit dampers, and **b**,**c** flag-shaped dissipators having **b** $\beta _F = 0.8$, and **c** $\beta _F = 0.6$

Using the empirical data from the IDA curves, a lognormal distribution is fitted to produce a collapse fragility for each building. Figure 10 shows the collapse fragility of three RC-CLT hybrid building designs. The spectral acceleration corresponding to the first mode $Sa(1.17 \text { s})$ is chosen as the intensity measure. Table 4 presents the fragility parameters. The fragility functions reaffirm the better seismic performance of buildings with flag-shaped dissipators, whose median collapse capacity is 25–30% more than those with slit dampers. Accordingly, the collapse margin ratio (*CMR*) defined as the ratio of median collapse capacity to the MCE level spectral acceleration is found to be 2.8 in the case of slit dampers, whereas it increases to 3.6 and 3.5 for buildings with flag-shaped dissipators. It is noted that an increase in the recentering ratio $\beta _F$ results in higher collapse capacity. This can be attributed to the larger hysteresis loops for $\beta _F=0.8$ compared to that for $\beta _F=0.6$, as shown earlier in Fig. 4. Thus, the flag-shaped dissipator with a higher recentering ratio provides higher energy dissipation and thus less deformation.

### Residual deformation

**Fig. 11:**
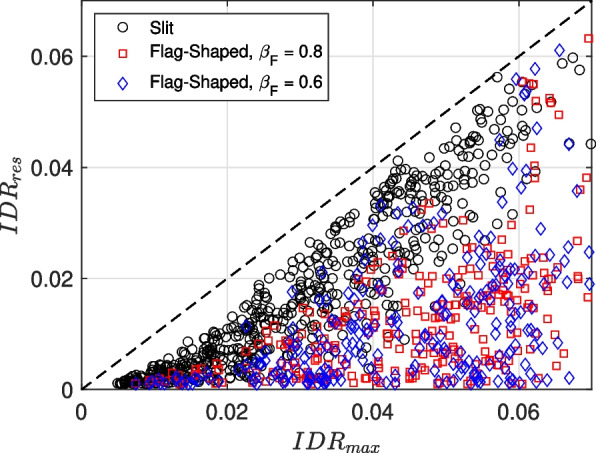
Maximum interstory drift ratio ($IDR_{max}$) versus the residual interstory drift ratio ($IDR_{res}$) for the RC-CLT hybrid building with different energy dissipators. The broken line represents the 45$^{\circ }$ case for equal maximum and residual values

While the maximum interstory drift ratio ($IDR_{max}$) is a useful measure for the collapse assessment of structures, the post-earthquake safety of a structure is largely determined based on the residual drift ratio ($IDR_{res}$). The literature is rich with studies on $IDR_{res}$ for single- and multi-degree-of-freedom systems (e.g., [7, 84–86]). Figure 11 shows a plot of the residual drift ratio versus maximum drift ratio for all three energy dissipators. It is observed that flag-shaped dissipators significantly reduce the residual drift. Thus, the recentering capacity of flag-shaped dissipators improves the building performance by reducing residual drift.

### Collapse risk assessment

The fragility functions developed in the previous section capture record-to-record uncertainty ($\beta _{RTR}$) in the buildings’ seismic performance. Additional sources of uncertainty were approximated based on the literature [87]. These include (a) uncertainty in design requirements, $\beta _{DR} = 0.10$: *Superior* rating on account of capacity-based RC frame obtained using DDBD, (b) uncertainty in test data, $\beta _{TD} = 0.20$: *Good* rating due to well-calibrated material and damper properties, and (c) modeling uncertainty, $\beta _{MDL} = 0.35$: *Fair* rating due to modest confidence in the representation of the collapse characteristics. Different uncertainty components are combined using the square root of the sum of their squares. Table 4 summarizes different measures of collapse for the RC-CLT hybrid building with energy dissipators. The probability of collapse at MCE, *P*(*coll*|*MCE*), for the building with slit dampers is found to be 2.7%. As anticipated from the fragility, the value of *P*(*coll*|*MCE*) for buildings with flag-shaped dissipators is smaller and equals to 1.7% and 1.9% for $\beta _F = 0.8$ and $\beta _F = 0.6$, respectively. These values are favorably compared to the target of 10% in code-compliant ordinary buildings based on ASCE 7 [88]. The collapse rate, $\lambda _{coll}$ is estimated using the total probability theorem considering all possible intensity levels. The value of $\lambda _{coll}$ is assessed as $0.65 \times 10^{-4}$ for the hybrid building with slit dampers, whereas $\lambda _{coll}$ for buildings with flag-shaped dissipators is found to be $0.42 \times 10^{-4}$ and $0.46 \times 10^{-4}$. These values again compare favorably to the collapse risk target of $2\times 10^{-4}$ in ASCE 7 [88]. Assuming a Poisson’s distribution for collapse, the probability of collapse in 50 years $P(coll)_{50y}$ is determined as 0.32% for the slit damper case; $P(coll)_{50y}$ takes the value of 0.21% and 0.23% for the cases with flag-shaped dissipators. These values again comfortably meet the conventional target of 1% for ordinary buildings [88].

**Table 4:** Collapse fragility parameters and associated seismic risk for the RC-CLT hybrid building with different energy dissipators

Ground Motion Suite	$\mu _{Sa}$ (g)	$\beta _{RTR}$	*CMR*	*P*(*coll*|*MCE*)	$P(coll)_{50y}$	$\lambda _{coll}$ ($\times 10^{-4}$)
Steel slit damper	1.103	0.335	2.8	2.7%	0.32%	0.65
SMA ($\beta _F$ = 0.8)	1.435	0.443	3.6	1.7%	0.21%	0.42
SMA ($\beta _F$ = 0.6)	1.387	0.443	3.5	1.9%	0.23%	0.46

## Conclusions

A global call for environmentally-sustainable construction is making timber an attractive material for building structural systems. The seismic performance of a hybrid timber building located in Vancouver, Canada is studied in this paper. The latest 6th generation seismic hazard model described in the NBCC 2020 coupled with the complex seismotectonic features of southwestern British Columbia make a compelling case for the present investigation. The six-story RC-CLT hybrid building contains steel slit dampers that enhance its energy dissipation mechanism. A hazard-consistent suite of 40 ground motion records representing three tectonic regimes (Subduction, Shallow Crustal, and In-slab) is selected for nonlinear response history analysis. The effectiveness of the energy dissipation mechanism using slit dampers is compared to a modern flag-shaped dissipator. Two flag-shaped dissipators are considered to observe the effect of different recentering ratios. The seismic performance of three building configurations is compared in terms of residual drift ratio. Flag-shaped dissipators are shown to outperform the slit dampers. The following conclusions are drawn from the study:

- The CLT infill and steel slit dampers in the building enhance its maximum base shear capacity by $\approx$ 70% compared to the bare RC frame. While the yield displacement of the hybrid building is marginally smaller due to the stiffer CLT infills, the maximum deformation capacity is enhanced by steel slit dampers. Thus, a combination of CLT infills and steel slit dampers increased both the lateral strength and drift capacity of the building.
- Comparable nonlinear static performance is obtained for the hybrid buildings with flag-shaped dissipators and steel slit dampers. However, when the nonlinear response history is compared, the buildings with flag-shaped dissipators outperform the ones with slit dampers.
- The median collapse capacity of the buildings with flag-shaped dissipators is 25–30% more than those with slit dampers. Correspondingly, the collapse margin ratio (*CMR*) is found to be 2.8 in the case of slit dampers, whereas it increases to 3.5–3.6 for buildings with flag-shaped dissipators.
- With an increase in the $\beta _F$ value, the flag-shaped hysteresis provides a better energy dissipation mechanism.
- Compared to the steel slit damper, the flag-shaped dissipators significantly reduce the residual drift under seismic excitation.
- The probability of collapse at the MCE for the hybrid building with slit and flag-shaped dissipators ($\beta _F=0.8$ and $\beta _F=0.6$) was found to be 2.7%, 1.7%, and 1.9%, respectively. These values are deemed favorable when compared to the 10% target for code-compliant ordinary buildings in ASCE 7 [88]. Further, the probability of collapse of the hybrid building in 50 years was computed as 0.32%, 0.21%, and 0.23%. These values again outperform the conventional target of 1% for ordinary buildings.

## Data Availability

The analysis models used in the current study are available from the corresponding author on reasonable request.

## Abbreviations

CLT: Cross-Laminated Timber
CMS: Conditional Mean Spectrum
CS: Conditional Spectrum
DDBD: Direct Displacement-Based Design
EVD: Equivalent Viscous Damping
IDA: Incremental Dynamic Analysis
IDR: Inter-Story Drift Ratio
IM: Intensity measure
NBCC: National Building Code of Canada
PSHA: Probabilistic Seismic Hazard Analysis
SHM6: 6th Generation Seismic Hazard Model
RC: Reinforced Concrete
